# Temperature and zooplankton size structure: climate control and basin-scale comparison in the North Pacific

**DOI:** 10.1002/ece3.1408

**Published:** 2015-01-31

**Authors:** Sanae Chiba, Sonia D Batten, Tomoko Yoshiki, Yuka Sasaki, Kosei Sasaoka, Hiroya Sugisaki, Tadafumi Ichikawa

**Affiliations:** 1 Research and Development Center for Global Change, JAMSTEC3173-25 Showamachi, Kanazawaku, Yokohama, Kanagawa, 2360001, Japan; 2 Sir Alister Hardy Foundation for Ocean Science4737 Vista View Cres., Nanaimo, BC, V9V 1N8, Canada; 3 Suidosha Co. Ltd.8-11-11 Ikuta, Tamaku, Kawasaki, Kanagawa, 2140038, Japan; 4 Fisheries Research Agency15F Queen's Tower B, 2-3-3 Minato Mirai, Nishiku, Yokohama, Kanagawa, 220-6115, Japan; 5 Fisheries Research Agency, National Research Institute of Fisheries Science2-12-4 Fukuura, Kanazawaku, Yokohama, Kanagawa, 2368648, Japan

**Keywords:** Climate control, continuous plankton recorder, copepod, functional diversity, North Pacific, Pacific decadal oscillation, thermal niche, zooplankton size

## Abstract

The global distribution of zooplankton community structure is known to follow latitudinal temperature gradients: larger species in cooler, higher latitudinal regions. However, interspecific relationships between temperature and size in zooplankton communities have not been fully examined in terms of temporal variation. To re-examine the relationship on a temporal scale and the effects of climate control thereon, we investigated the variation in copepod size structure in the eastern and western subarctic North Pacific in 2000–2011. This report presents the first basin-scale comparison of zooplankton community changes in the North Pacific based on a fully standardized data set obtained from the Continuous Plankton Recorder (CPR) survey. We found an increase in copepod community size (CCS) after 2006–2007 in the both regions because of the increased dominance of large cold-water species. Sea surface temperature varied in an east–west dipole manner, showing the typical Pacific Decadal Oscillation pattern: cooling in the east and warming in the west after 2006–2007. The observed positive correlation between CCS and sea surface temperature in the western North Pacific was inconsistent with the conventional interspecific temperature–size relationship. We explained this discrepancy by the geographical shift of the upper boundary of the thermal niche, the 9°C isotherm, of large cold-water species. In the eastern North Pacific, the boundary stretched northeast, to cover a large part of the sampling area after 2006–2007. In contrast, in the western North Pacific, the isotherm location hardly changed and the sampling area remained within its thermal niche throughout the study period, despite the warming that occurred. Our study suggests that while a climate-induced basin-scale cool–warm cycle can alter copepod community size and might subsequently impact the functions of the marine ecosystem in the North Pacific, the interspecific temperature–size relationship is not invariant and that understanding region-specific processes linking climate and ecosystem is indispensable.

## Introduction

Zooplankton functional diversity, estimated from its trophic structure and size composition, is closely related to ocean productivity and biogeochemical processes. Understanding the spatial and temporal variability of zooplankton functional diversity and the mechanism(s) underlying this variability is indispensable in assessing the impacts of prevailing and future environmental threats, such as ocean warming and acidification, on marine ecosystem functions, to sustain marine ecosystem services.

Zooplankton size is particularly important because it can be an indicator of multiple biological and ecological traits, such as metabolism, feeding strategy (Kiørboe 2011), and trophic links (Barton et al. 2013). In a meta-analysis of temperature–size relationships at various levels of organization from the individual organism to the community in aquatic ecosystems, Daufresne et al. (2009) reviewed species-shifts induced by temperature change, for example, dominance of large versus smaller species could result in temporal variation in average body size of plankton and fish communities. Researchers have investigated the influences of such interspecific variation in copepod community size on regional fisheries productivity (Beaugrand and Kirby, 2010; Bi et al. 2011) and vertical carbon transport efficiency (Beaugrand et al.*,* 2010) using long-term monitoring data sets. The canonical distribution pattern of temperature and copepod species composition is widely recognized that larger species dominate in cooler, higher latitudinal regions and smaller species dominate in warmer, lower latitudinal regions (San Martin et al. 2006). Compared with smaller, warmer-water species, larger, colder-water species generally enhance the production of higher trophic levels because of the higher lipid content (Hooff and Peterson 2006) and in the efficiency of vertical carbon transport (Kobari et al. 2003). Thus, the recently observed high-latitudinal shift of the distribution of warmer-water species associated with regional warming trends (Beaugrand et al. 2002; Peterson and Schwing 2003; Mackas et al. 2007) is of concern as a potential precursor of changes in biological productivity and biogeochemical cycles in a future warming environment.

However, other time series studies have reported results that seemingly contradict the global interspecific temperature–copepod size relationship: dominance of larger, colder-water species in warmer years (Tadokoro et al. 2005; Chiba et al. 2006; Robinson et al. 2014), indicating that the temperature–size relationship is not always consistent in terms of temporal variation or regional zooplankton communities. Temperature alters the rates of various biological processes in copepods, such as their growth, productivity, and mortality (Hirst and Kiørboe 2002), and influences seasonal cycles of physical and chemical properties, such as stratification and nutrient availability in the upper water column, which determines phytoplankton, and subsequently zooplankton, size structures through size-dependent prey–predator interactions (Hansen et al. 1994) and region-specific trophic amplification (Chust et al. 2014). Thus, the discrepancies found in those previous studies raise the question as to what regionally specific physical, chemical, and biological mechanism(s) mediate the observed relationships between temperature and copepod community structure.

The eastern and western subarctic North Pacific differ in terms of their physical, chemical, and biological properties and biogeochemical processes, such as the extent of winter-time mixing, nutrient availability, and phytoplankton seasonality (Harrison et al. 2004; Sasaoka et al. 2011). Long-term ecosystem change studies in the North Pacific have revealed an east–west contrast in lower trophic-level responses to environmental variability, controlled by decadal climate dynamics, such as the Pacific Decadal Oscillation (PDO) (Di Lorenzo et al. 2014). The North Pacific Continuous Plankton Recorder (CPR) survey has conducted longitudinal transect surveys since 2000 over the subarctic North Pacific; the survey area covers the regions of the Gulf of Alaska, Western Subarctic Gyre, and Oyashio (Batten et al. 2006). Despite the east–west contrast in environments, copepod species composition is roughly similar in the oceanic domain of these regions (Mackas and Tsuda 1999). Copepod samples collected through the North Pacific CPR transect thus provide an ideal data set for regional comparisons of variation in copepod community structure in conjunction with the basin-scale climatic effects thereon.

Using 12 recent yearly CPR data sets, we re-examined the conventional view on the global distribution of interspecific temperature–size relationship for copepods community in the subarctic North Pacific, and sought to reveal region-specific mechanisms linking climate to the spatiotemporal variation in copepod community structure.

## Materials and Methods

### Observation and data

**Figure 1:**
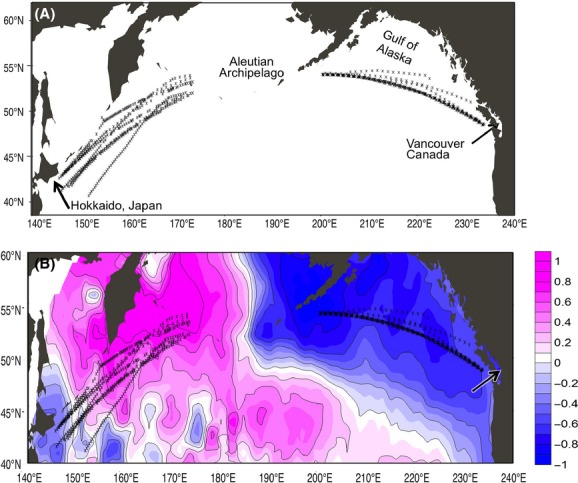
(A) Sampling locations of the summertime transect (June and July) of the Continuous Plankton Recorder (CPR) survey 2000–2011 in the Western region (40–55°N, 140–175°E) and Eastern region (45–55°N, 200–235°E). (B) Normalized difference in the June–July average sea surface temperature between 2007–2011 and 2000–2005 with the location of each CPR sampling (asterisk) superimposed.

Zooplanktons were collected in the Continuous Plankton Recorder (CPR) survey from 2000 to 2011. CPRs were regularly towed along the approximate by volunteer ships three times per year – spring, summer, and autumn – along transects between Vancouver and Hokkaido (Fig.1A). Detailed information of the North Pacific CPR survey can be found in Batten et al. *(*2006). In this study, we used only summer-time samples obtained during June and July because interannual variation in the sampling location and date are minimal in summer-time transects. We analyzed samples obtained in areas of the western North Pacific (40–55°N, 143–170°E) and eastern North Pacific (45–55°N, 123–160°W). As samples between the two regions were collected along the Aleutian Archipelago, we did not analyze them to avoid the possible influence of coastal environmental signals on zooplankton composition (Batten et al. 2006).

Copepods in each sample were identified microscopically to the species or genus levels, some of which were further divided into copepodite stages, and the number of individuals per sample was estimated for each of 52 taxa (Table1). Detailed handling and analytical methods of CPR zooplankton samples are described in Batten et al. (2003) and Richardson et al. (2006). The distance covered by a single sample is 18.5 km, and every other sample and every fourth sample were analyzed for the western and eastern regions, respectively, following a fully standardized methodology.

**Table 1:** List of copepod taxonomic categories with respective total length of adult female (mm), optimal sea surface temperature (SST) (°C), and the categorized SST-size functional groups. Optimal SST is defined as the mode SST of occurrence of each species. Functional groups are defined by optimal SST and total length, CWS (cold-water small) species: total length <4.0 mm, optimal SST <9.0°C; CWL (cold-water large) species: total length >4.0 mm, optimal SST <9.0°C, WWS (warm-water small) species: total length <4.0 mm, optimal SST >9.0°C (see Fig.6 on categorizing criteria of the functional groups)

Taxonomic category	Total length of Adult female (mm)	Optimal SST (°C)	Functional Group
*Acartia danae*	1.2	4.8	CWS
*Acartia longiremis*	1.2	10.2	WWS
*Acartia* spp.	1.2	8.1	CWS
*Acartia tumida*	2.0	7.3	CWS
*Calanus jashnovi*	3.9	14.2	WWS
*Calanus marshallae*	4.0	5.3	CWL
*Calanus pacificus*	2.9	11.0	WWS
*Calanus* spp. CI_CIV	2.9	7.9	CWS
*Calanus* spp. CV-VI	2.9	15.7	WWS
*Candacia colombiae*	4.2	6.2	CWL
*Candacia pacifica*	4.2	4.5	CWL
*Centropages abdominalis*	1.4	11.1	WWS
*Clausocalanus* spp.	1.1	8.3	CWS
*Corycaeus* spp.	1.2	15.0	WWS
*Ctenocalanus vanus*	1.1	14.3	WWS
*Eucalanus bungii*	7.3	6.3	CWL
*Eucalanus elongatus*	6.5	3.3	CWL
*Eucalanus pileatus*	2.2	15.2	WWS
*Eucalanus* spp.	4.9	6.1	CWL
*Euchaeta* spp.	3.1	8.6	CWS
*Euchirella rostrata*	2.8	7.9	CWS
Harpacticoida spp.	1.0	6.2	CWS
*Heterorhabdus* spp.	3.3	9.9	WWS
*Heterorhabdus tanneri*	3.8	5.6	CWL
*Labidocera* spp.	2.4	15.5	WWS
*Mesocalanus tenuicornis*	2.5	16.7	WWS
*Metridia okhotensis*	4.7	5.9	CWL
*Metridia pacifica*	3.1	8.6	CWS
*Metridia* spp. CV-CVI	3.1	7.4	CWS
*Metridia* spp. I-IV	3.1	7.9	CWS
*Microcalanus* spp.	0.9	12.0	WWS
*Neocalanus cristatus* CI-CIV	8.9	3.7	CWL
*Neocalanus cristatus* CV_CVI	8.9	6.4	CWL
*Neocalanus flemingeri*	4.7	4.7	CWL
*Neocalanus plumchrus* (stage unidentified)	5.3	5.3	CWL
*Neocalanus plumchrus* CII	5.3	6.7	CWL
*Neocalanus plumchrus* CIII	5.3	8.4	CWL
*Neocalanus plumchrus* CIV	5.3	7.0	CWL
*Neocalanus plumchrus* CV	5.3	8.1	CWL
*Neocalanus* spp. (stage unidentified)	5.3	5.4	CWL
*Oithona* spp.	1.3	7.7	CWS
*Oncaea* spp.	1.1	8.7	CWS
*Paracalanus* spp.	1.1	14.3	WWS
*Paraeuchaeta elongata*	6.1	6.3	CWL
*Paraeucheata* spp.	7.6	7.7	CWL
*Paraheterorhabdus robustus*	4.1	7.4	CWL
*Pleuromamma* spp.	3.2	8.4	CWS
*Pseudocalanus* spp.	1.2	8.5	CWS
*Pseudolubbockia dilatata*	2.9	3.1	CWS
*Rhincalanus nasutus*	3.3	9.6	WWS
*Scolecithricella* spp.	1.3	5.7	CWS
*Temora discaudata*	1.8	15.2	WWS

To investigate the climate and environmental drivers that affect interannual and regional variation in copepod size structure, we used satellite sea surface temperature data. Spatial and temporal sea surface temperature anomalies are well documented as indicators of basin-scale climate–ocean interaction over the subarctic North Pacific, such as the Aleutian Low-Pacific Decadal Oscillation (PDO) system (Di Lorenzo et al. 2014). Advanced very high resolution radiometer (AVHRR) pathfinder level 3 sea surface temperature data (ver. 5) for 2000–2009 were obtained from the NASA Jet Propulsion Laboratory – Physical Oceanography Distributed Active Archive Center (JPL–PO.DAAC) website (http://podaac.jpl.nasa.gov/dataset/AVHRR_PATHFINDER_L3_SST_DAILY_DAYTIME_V5?ids=&values=&search=Pathfinder), and Microwave - Infrared Optimally Interpolated SST data (MR-IR OI SSTs, ver. 4) for 2010–2011 were obtained from the Remote Sensing Systems website (http://www.remss.com/measurements/sea-surface-temperature). Original daily averaged sea surface temperature data at a 4-km resolution were re-gridded to 1°×1 resolution to obtain sufficient data coverage with at least one datum point per grid, and the 10-day average of the gridded sea surface temperature was matched to the date and location of each sample. The average sea surface temperature of the month and the previous month for each zooplankton sampling, and the spring-time (March–May) average sea surface temperature at each sampling location grid were also estimated.

### Analysis

For each sample with a total copepod abundance of more than 50 individuals, we estimated the average copepod community size (CCS) as an indicator of zooplankton functional diversity (Richardson et al. 2006) (Table1). We excluded samples with a smaller total abundance to avoid including skewed and/or extreme values. Copepod community size was estimated based on the total length of adult females and the abundance of respective species following the formula below (Richardson et al. 2006),

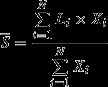


where *L*_*i*_ (mm) is the female total length and *X*_*i*_ is the abundance (number of individuals per samples) of each copepod species *i*, respectively, and N is the overall copepod abundance. Because CCS is based on the adult female size of each species regardless of the developmental stage of the observed individual copepod, CCS indicates species composition, such as dominance of larger versus smaller copepods, rather than the actual size composition of the copepod community. The total length of adult females for each species is defined following the description in (Chihara and Murano 1997) and online references (http://copepodes.obs-banyuls.fr/en/fichesp.php). When individuals were identified only to the genus level, the size of the most frequently occurring species of the respective genus was assumed; for example, *Calanus* spp. were assumed to be *Calanus pacificus*, so 2.9 mm was given as its total length. The CCS values were compared with the 10-day average sea surface temperature of the grid that matched the sampling day and location. The average sea surface temperature of the month, preceding month, and preceding season (March–May) of the sampling date at the same grid were also compared with the CCS to assess whether the environmental conditions preceding the sampling influenced the summer-time copepod community structure.

As the sampling depth of the CPR is approximately 10 m and zooplanktons were continuously collected day and night, we examined the possible bias in CCS, which might be caused by species with a diel vertical migration such as *Metridia* spp.. However, there was no significant difference in average CCS of night samples and day samples in any year. Although night and day samples were alternately collected every 4–10 samples every year, there were no such day and night cycles detected in the CCS (Fig. S1).

To investigate the species-specific thermal niche and its influence on regional copepod community structures and the observed CCS variation, the optimal sea surface temperature at which a taxon was most abundant was estimated for each of 52 taxa using the following formula,

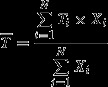


where *T*_*i*_ (in °C) is the sea surface temperature on the date and location of sampling, *X*_*i*_ is the abundance (number of individuals per samples) of each copepod species *i*, and N is the overall copepod abundance. We classified the copepod taxa into functional groups in terms of the respective optimal temperatures and sizes (total length of adult female) and examined interannual variation in the abundance of each group.

### Correction for sampling bias

Although the sampling locations and dates remained relatively constant throughout the research period in the summertime samples, possible sampling bias in the interannual variation of time series should be eliminated as much as possible. To examine such influences, stepwise multiple linear regression was conducted on CCS, sea surface temperature, and the abundance of each copepod functional group with latitude, longitude, and Julian day as independent variables (Table S1) to model the base values expected from the respective sampling date and location of each time series. We defined the residuals of the observed values minus the base values as the corrected CCS, sea surface temperature, and abundance (Fig. S1 shows an example of CCS correction in the western region). Annual averages were estimated based on those residuals. No correction of the data used for estimation of the optimal temperature for each copepod species was performed.

## Results

### Copepod community size and sea surface temperature

**Figure 2:**
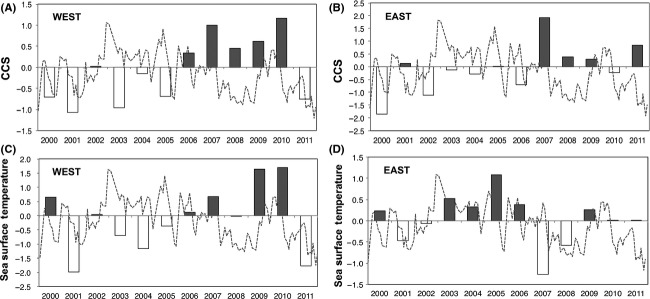
Interannual variation of copepod community size (CCS) in the Western region (A) and the Eastern region (B), and of sea surface temperature in the respective regions (C, D). The values are annual averages of the corrected CCS and sea surface temperature. Dotted line is Pacific Decadal Oscillation index (monthly) for 2000-2011 (http://jisao.washington.edu/pdo/).

**Figure 3:**
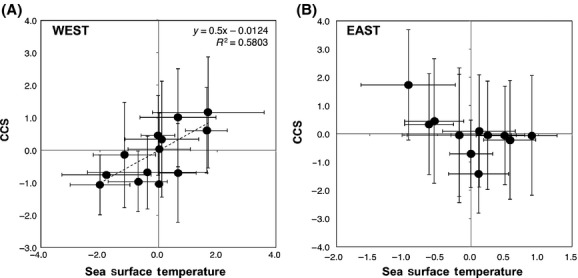
Relationship between interannual variations of copepod community size (CCS) and sea surface temperature in the Western and Eastern regions during 2000-2011. Each dot and error bar indicates the annual average and standard deviation, respectively, based on the corrected CCS and sea surface temperature (see Fig.2).

Annual average CCS increased generally after 2007 in the eastern North Pacific and 2006 in the western North Pacific, roughly coinciding with the phase shift of the Pacific Decadal Oscillation index (Fig.2A, B). The contrast in CCS before and after 2006 was particularly conspicuous in the West. Anomalies of the 10-day averaged sea surface temperature at the sampling times and sites also changed in 2006–2007 in both the East and West, but in an opposite manner between the regions: a decrease in the East versus an increase in the West (Fig.2C, D). We found a significant positive correlation between interannual sea surface temperature and CCS in the West (*R*^2^ = 0.5803, *P* = 0.004; Fig.3) indicating greater dominance of larger copepod species in warmer years. In contrast, there was no significant relationship between the sea surface temperature and CCS in the East (*P* = 0.056), although larger species tended to dominate in cooler years. In both regions, the monthly and seasonal average sea surface temperatures showed weaker correlations with CCS than the 10-day average sea surface temperature, which covered the sampling date (data not shown).

To assess the spatiotemporal variation of sea surface temperature over the subarctic North Pacific, particularly before and after 2006/2007 when the CCS increased in both regions, we compared the mean sea surface temperatures (June–July) for 2000–2005 and 2007–2011 in the areas of 40–60°N and 140°E–123°W. The sea surface temperature anomaly map showed cooling and warming patterns over the sampling areas in the East and West in the latter period (Fig.1B), suggesting that the observed interannual sea surface temperature variation was likely derived from basin-scale climate–ocean dynamics rather than smaller, local-scale events.

### Thermal niche and size groups

**Figure 4:**
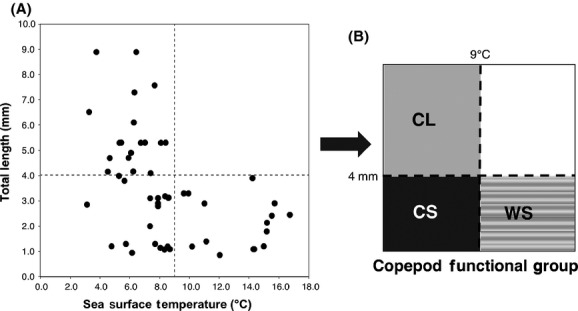
(A) Relationship of total length and optimal sea surface temperature of 52 copepod taxa (see Table1). Each dot indicates the total length (mm) of adult females of the respective species and the mode sea surface temperature at which the highest abundance occurred. (B) Classification of copepod functional groups by the sea surface temperature (9°C) and total length (mm) criteria: cold-water large (CL), cold-water small (CS), and warm-water small (WS).

The relationship between the optimal sea surface temperature and size (total length of adult females) in 52 taxa was found to be negative: Larger (smaller) species were more abundant in cooler (warmer) conditions (Fig.4A). In particular, the optimal sea surface temperatures of the taxa larger than 4.0 mm were exclusively below 9°C. According to these size-sea surface temperature criteria, we classified copepods taxa into the following three functional groups: cold-water large (CL: size >4.0 mm, sea surface temperature <9°C), cold-water small (CS: size <4.0 mm, sea surface temperature <9.0°C), and warm-water small (WS: size <4.0 mm, sea surface temperature >9.0°C) groups (Table1, Fig.4B).

**Figure 5:**
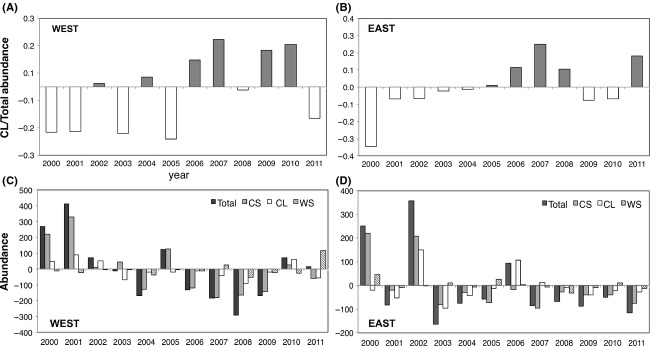
Anomalous ratio of cold-water large species (CL) abundance to total copepods abundance in the Western region (A) and Eastern region (B). Anomalous abundances (individuals per sample) of total copepods, CL, cold-water small species (CS) and warm-water small species (WS) in the Western region (C) and Eastern region (D).

Interannual variation of the relative abundance of CL to the total copepod abundance showed a similar pattern to that of CCS: higher after 2006 in both the East and West (Fig.5A, B), suggesting that the relative dominance of CL against CS and WS determined the interannual CCS variation. However, the variation in the total abundances differed from those in the CL ratio and CCS: higher in the early 2000s and remained at a low level until 2011 in both regions (Fig.5C, D). In both regions, the abundance of CS and CL varied generally in a manner similar to the total abundance; however, the CS were more abundant and better explained the variation in total abundance (Spearman's *R* = 0.790, *P* < 0.01 in the East; *R* = 0.944, *P* < 0.01 in the West) than did the CL (Spearman's *R* = 0.427, n.s. in the East; *R* = 0.692, *P* < 0.05 in the West). WS abundance comprised a relatively small fraction (<6%), with the exception of in 2011 in the West and in 2005 and 2008 in the East, and thus least explained the interannual variation in total abundance.

**Figure 6:**
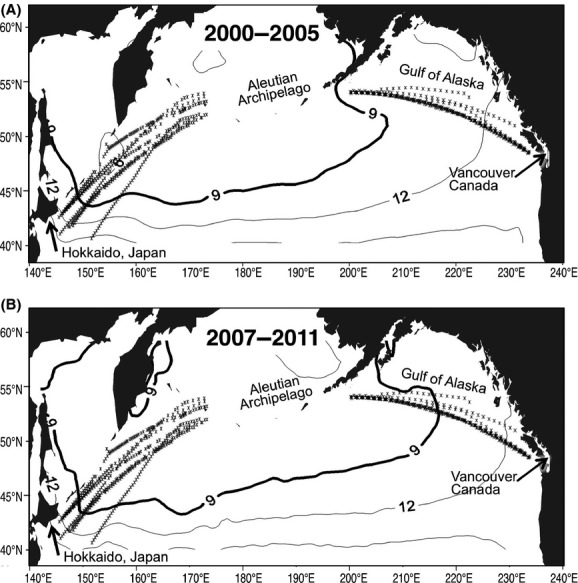
Comparison of the average location of the 9°C isothermal in June and July during 2000-2005 (A) and 2007-2011 (B). Locations of Continuous Plankton Recorder sampling (asterisk) are superimposed.

Assuming that a sea surface temperature of 9°C indicates the boundary of the thermal niche of the CL group, from the information in Figure4, we compared the geographical locations of the 9°C isotherm between 2000–2005 and 2007–2011 (Fig.6). In the East, while the CPR survey area was located exclusively in an area warmer than 9°C during 2000–2005, almost half of it was enclosed in an area cooler than 9°C in 2007–2011 as the 9°C boundary stretched northeastward. In the West, however, the 9°C boundary locations were almost identical between the two periods, and ∽80% of the CPR survey area remained within the area cooler than 9°C both before and after 2006–2007.

## Discussion

### Variability in the temperature–size relationships

The increase in CCS in warmer years in the West is contrary to the conventional theory, suggesting that warmer conditions favor dominance of smaller species (Daufresne et al. 2009) (Fig.2). From the observed overall negative relationship between the optimal temperature and size of individual copepods species, indicating that cooler (warmer) conditions favor larger (smaller) copepods (Fig.4), we conclude that the canonical distribution pattern of copepods size structure does likely follow the latitudinal temperature gradient in the North Pacific, as suggested by the previous study (San Martin et al. 2006). However, our study also suggests that careful consideration is needed when applying the conventional interspecific temperature–size relationship in copepod community (Daufresne et al. 2009), especially to the mechanism of its temporal variation.

Given that the relative dominance of the CL group was responsible for interannual variation of CCS in both regions (Fig.5) and that those species; for example, *Neocalanus* spp. and *Eucalanus bungii*, are dominant in both regions (Mackas and Tsuda 1999), the observed positive relationship between CCS and temperature in the West may seem counterintuitive. We demonstrated that this discrepancy in the interspecific temperature–size relationship could be explained by a geographical shift of the thermal niche boundary of these species (Fig.6). Here, we discuss possible mechanisms in copepod community responses to spatiotemporal temperature variation and their differences in the eastern and western subarctic North Pacific.

In the West, temperature increases in the mid-2000s occurred within the thermal niche of the CL species and apparently had no negative impact on these species. Although our study detected no abundance increase in the CL group associated with warming, significant abundance and/or biomass increase in those species in warmer years has been reported by decadal-scale studies in the western subarctic North Pacific. For example, the annual biomass of *Neocalanus* spp. increased and contributed to a marked increase in total zooplankton biomass during the 1990s when warming conditions prevailed (Tadokoro et al. 2005; Chiba et al. 2006). The increase in copepod biomass during the 1990s was attributed to the “good match” with the early occurrence of phytoplankton bloom timing induced by warming (Chiba et al. 2008). This phenology-driven, bottom-up control hypothesis was supported by nitrogen stable isotope analysis, and its positive impact on salmon production was reported (Chiba et al. 2012b). A study of satellite-derived Chl *a* additionally revealed that spring bloom occurred with earlier timing in the late 2000s (Chiba et al. 2012a). Dominant CL species, for example, *Neocalanus* spp. and *Eucalanus bungii*, develop in the surface layer from spring to summer, and thus, spring food availability is likely to influence their springtime population growth and consequently summertime abundance. The early spring bloom might be responsible for dominance of the CL group in the warmer years as observed in the 1990s.

In our study, the thermal niche boundary, 9°C, was determined based on sea surface temperature at sampling sites and did not necessarily reflect any physiological thermal window (e.g., Alcaraz et al. 2014) of the copepod species. Moreover, vertical distribution ranges of some copepod species listed in Table1 are deeper than the CPR sampling depth of 10 m, and the actual temperature range of their habitats are unknown. It is not possible to estimate to what extent the warming in the late 2000s directly affected copepod physiology. We should rather consider the thermal niche boundary, 9°C, as the indicator of spatiotemporal variation in the water column environments, which determined regional nutrients-light conditions within the mixed layer and thus influenced phytoplankton phenology. Observing the positive relationship between copepod community size and water temperature in the Southern Ocean, Robinson et al. (2014) also pointed out the bottom-up control driven by the mixed-layer process rather than a direct influence of temperature as the mechanism of the observed counter-intuitive temperature–size relationship. In summary, it is suggested that CL abundance and CCS in the western North Pacific temporally vary through bottom-up control driven by a seasonal mixed-layer processes. We were unable to examine other factors such as temperature influence on predation pressure on the CL species without additional data.

In the East, the thermal niche boundary for the CL species shifted northeastward toward the sampling area in the mid-2000s, when a CCS increase was observed. Although the negative relationship between interannual variation of CCS and sea surface temperature was not significant (Fig.3B), this might be because ∽60% of the sampling area was outside the boundary (Fig.6). In contrast to the West, the observed relationship between the temperature and CCS was negative: cooler-larger and warmer-smaller. Changes in the dominance of cold-water and warm-water copepod species on a decadal scale associated with regional temperature anomalies have been reported in the eastern North Pacific, in the wide latitudinal areas from southern California to Oregon (Peterson and Schwing 2003), and the Gulf of Alaska (Mackas et al. 2007). The observed copepod size structure change was attributed to advection transport (Keister et al. 2011) and latitudinal shifts in oceanic boundaries (Batten and Freeland 2007), both of which are driven by oceanic current dynamics. Thus, the proposed mechanisms responsible for the regional cool–warm cycle and copepod community structure differ from those in the West.

One question remaining is why our study failed to find a response in the CS group to interannual sea surface temperature variation, while it apparently has the same thermal niche boundary (9°C) as the CL group. We found that CS tended to increase in cooler condition in both East and West, indicating more susceptibility for warming. The warm condition in the late 2000s induced not only earlier spring blooms but also earlier termination of the bloom; thus, the phytoplankton abundance in June–July was low compared to the cool years (Chiba et al. 2012b2012a). While the earlier spring bloom seems to benefit summertime abundance of the CL group with longer life cycles, the earlier termination of the bloom likely to have a negative impact on the CS group, because smaller species with short life cycle are more dependent on summertime phytoplankton availability. However, additional supporting data are needed to confirm this hypothesis.

### Climate control

Climate control related to the Pacific Decadal Oscillation (PDO) (Mantua and Hare 2002) is likely to be a major factor differentiating physical processes and subsequent responses in copepod community structure between the eastern and western North Pacific. The distribution pattern of sea surface temperature over the subarctic North Pacific, indicated by the PDO index (http://jisao.washington.edu/pdo/PDO.latest), is closely related to the Aleutian Low dynamics, which dominate wind stress and oceanic circulation over the North Pacific (Miller et al. 1994; Trenberth and Hurrell 1994). The observed changes in CCS and sea surface temperature in 2006–2007 coincided approximately with the timing of the negative to positive shift in the PDO index (Fig.2), indicating cooling in the eastern North Pacific and warming in the western North Pacific. The basin-scale sea surface temperature distribution before and after 2006 showed a clear east–west dipole pattern: the typical PDO pattern (Fig.1B). In a review of the mechanisms of climate–ocean interactions and their control of the lower trophic levels in the North Pacific, Di Lorenzo et al. (2014) showed that the Aleutian Low-PDO system controlled oceanic current dynamics and latitudinal shifts of oceanic fronts in the eastern North Pacific and seasonal mixed-layer processes, such as winter-time mixing and nutrient availability in the western North Pacific. Therefore, Aleutian Low-PDO influenced the lower trophic level through advection control in the east and through the bottom-up trophic interaction in the west, as described in the preceding section. This climate–ocean–ecosystem scenario might explain the east–west discrepancy in the extent of the geographical shift in the thermal niche boundary.

The basin-scale east–west contrast in zooplankton responses to a climate-controlled cool–warm anomaly is well documented in the subarctic North Atlantic. After the phase shift in the North Atlantic Oscillation (NAO) in the mid-1980s, the large cold-water species, *Calanus finmarchicus*, decreased in the North Sea with an increase in warm-water species and a rise in water temperature (Beaugrand et al. 2002). However, in the western North Atlantic, the NAO-ocean circulation interaction induced more complex responses in *C. finmarchicus* abundance that could not be explained simply by a temperature anomaly (Greene et al. 2003). These studies, together with our findings, indicate that understanding region-specific mechanisms linking climate to zooplankton functional diversity is essential for assessing the impact of observed or predicted global temperature variation on regional- to basin-scale marine ecosystems.

The sea surface temperature variations in our study areas did not follow the PDO index perfectly. For example, we did not observe cool (warm) conditions in the eastern (western) North Pacific in 2000–2002, as would be expected from the negative PDO signal. We also found no clear relationship between PDO and copepod abundance. In fact, changes in the abundance were more conspicuous before and after 2001–2002 rather than in 2006–2007 (Fig.5). Although Aleutian Low-PDO control functioned as a base mechanism for temperature–CCS during the period of our study, other climatic factors, such as the North Pacific Gyre Oscillation (NPGO) (Di Lorenzo et al. 2008), may have dominated the ocean–ecosystem interaction under certain conditions (Miller et al. 2014).

### Summary

We revealed basin-scale mechanisms linking climate to interspecific zooplankton size structure in the subarctic North Pacific for the first time using data obtained by fully comparative and standardized methods. With increasing demand for the establishment of a global ecosystem observation system, defining the most appropriate and practical plankton indicators, including zooplankton size structure at both intra- and interspecific levels, is vital (Racault et al. 2014). As scientific evidence on the temperature–zooplankton relationship and future projections of global warming scenarios (IPCC 2013) accumulates, the possible effects of future temperature increases on marine ecosystem productivity as a result of changes in zooplankton are under investigation (reviewed in Richardson 2008; Daufresne et al. 2009). However, these future scenarios have considerable uncertainties, partly because of heterogeneity in relationships between temperature and plankton traits, as observed in this study. To reduce the uncertainties in warming projections of the lower trophic levels on a basin-to-global scale, further comparative studies of various oceanic regions should be performed through networking of existing regional ecosystem monitoring programs, such as the Global Alliance of CPR Survey (GACS: http://www.globalcpr.org), as recommended by others (Edwards et al. 2010).

## Supporting Information

**Figure S1**. Correction of copepod community size (CCS) of the Western region.

**Table S1.** Stepwise Multiple Liner Regression on Copepod Community Size (CCS), Sea Surface Temperature (SST), abundance of each copepods category, and ratio of Cold water species to Total abundance with Latitude (Lat), Longitude (Long) and Julian Day (JD) as independent variables in the western and eastern North Pacific.
