# Supplementary figures and images for: Temperature and zooplankton size structure: climate control and basin-scale comparison in the North Pacific
Source: Ecol Evol. 2015 Jan 31;5(4):968–78. doi: 10.1002/ece3.1408 (PMC4338978; doi:10.1002/ece3.1408)

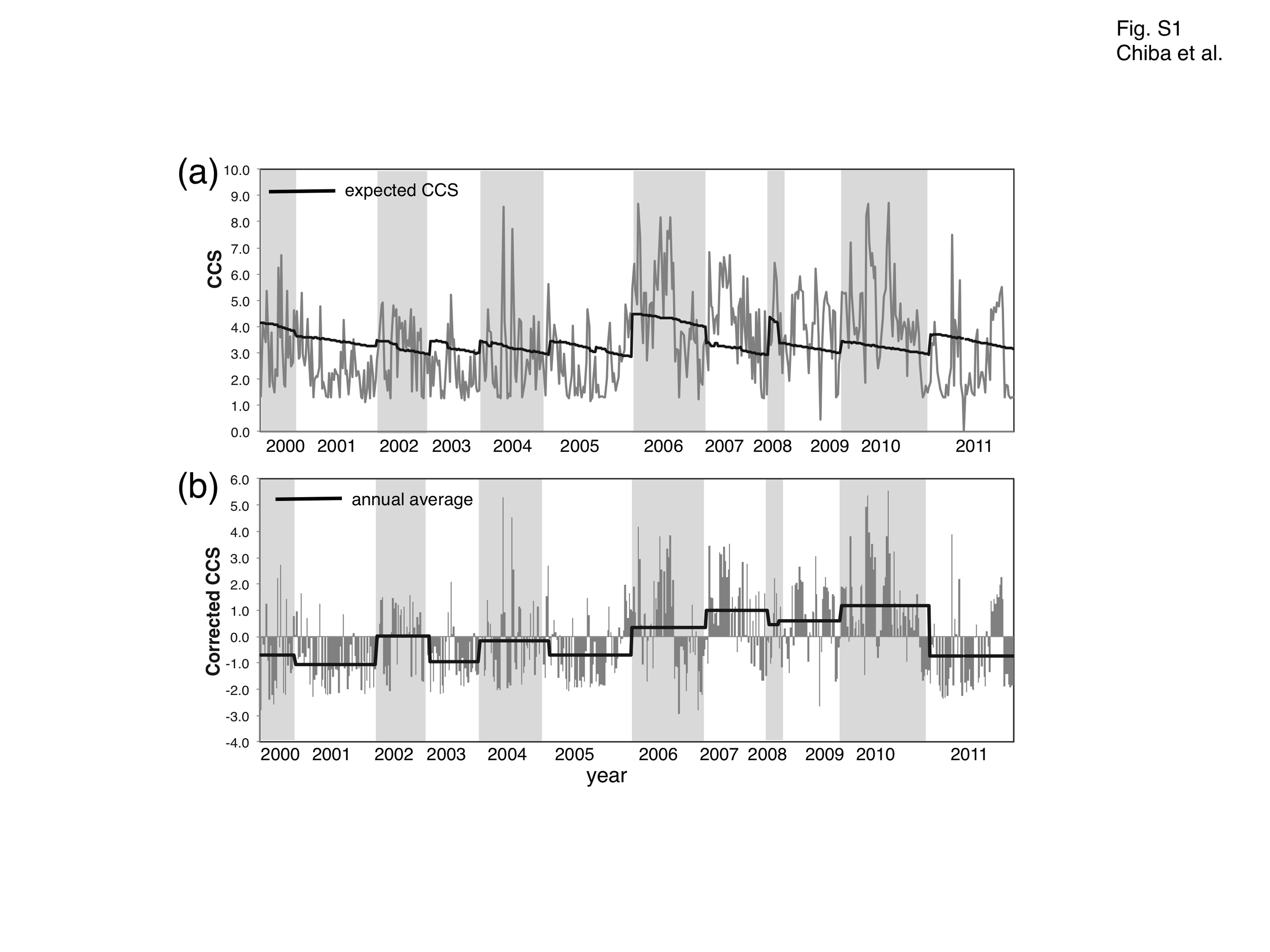

Supplement: Supplementary file 1 [file ece30005-0968-sd1.jpg]
