# Supplementary material for: Temperature and zooplankton size structure: climate control and basin-scale comparison in the North Pacific
Source: Ecol Evol. 2015 Jan 31;5(4):968–78. doi: 10.1002/ece3.1408 (PMC4338978; doi:10.1002/ece3.1408)
Supplement: Supplementary file 2 [file ece30005-0968-sd2.docx]

**Table S1.** Stepwise Multiple Liner Regression on Copepod Community Size (CCS), Sea Surface Temperature (SST), abundance of each copepods category, and ratio of Cold water species to Total abundance with Latitude (Lat), Longitude (Long) and Julian Day (JD) as independent variables in the western and eastern North Pacific. No significant regression for the ratio of Coldwater Large group to Total abundance in the eastern North Pacific was detected.

|  | Time Series | Regression | Adjusted R^2^ | P |
| --- | --- | --- | --- | --- |
| WEST | |  |  |  |
|  | CCS | 0.054 *Lat* + 0.027*JD* - 3.721 | 0.060 | 0.000 |
|  | SST | 0.386 *Long* + 0.121 *JD* - 1.357 *Lat* - 9.691 | 0.596 | 0.000 |
|  | Abundance of Coldwater Large Group | 1.938 *JD* - 169.042 | 0.009 | 0.011 |
|  | Abundance Coldwater Small Group | -21.438 *Lat* + 1350.919 | 0.018 | 0.001 |
|  | Abundance Warmwater Group | 1.169 *JD* + 10.133 *Long* - 27.672 *Lat* - 458.365 | 0.071 | 0.000 |
|  | Coldwater Large/Total abundance | 0.004 *JD* + 0.0015 *Lat* - 0.958 | 0.045 | 0.000 |
| EAST |  |  |  |  |
|  | CCS | 0.033 *Long* + 0.376 *Lat* - 22.777 | 0.025 | 0.000 |
|  | SST | -0.435 *Lat* + 0.075 *Long* + 0.050 *JD* + 7.338 | 0.873 | 0.000 |
|  | Abundance of Coldwater Large Group | -2.471 *JD* + 549.135 | 0.029 | 0.002 |
|  | Abundance Coldwater Small Group | -1.704 *Lat* - 2.917 *JD* +1026.89 | 0.022 | 0.014 |
|  | Abundance Warmwater Group | 0.567 *JD* -7.382 *Lat* + 312.689 | 0.049 | 0.000 |
|  | Coldwater Large/Total abundance | n. s. | - | - |
|  |  |  |  |  |
